# Buprenorphine Prescribing Characteristics Following Relaxation of X-Waiver Training Requirements

**DOI:** 10.1001/jamanetworkopen.2024.25999

**Published:** 2024-08-05

**Authors:** Paul J. Christine, Rouba A. Chahine, Simeon D. Kimmel, Nicole Mack, Christian Douglas, Thomas J. Stopka, Katherine Calver, Laura C. Fanucchi, Svetla Slavova, Michelle Lofwall, Daniel J. Feaster, Michael Lyons, Jerel Ezell, Marc R. Larochelle

**Affiliations:** 1 Division of General Internal Medicine, Department of Medicine, University of Colorado School of Medicine, Aurora; 2 Department of General Internal Medicine, Denver Health and Hospital Authority, Denver, Colorado; 3 Social, Statistical, and Environmental Sciences, Research Triangle Institute, Research Triangle Park, North Carolina; 4 Section of General Internal Medicine, Department of Medicine, Boston Medical Center, Boston, Massachusetts; 5 Section of General Internal Medicine, Department of Medicine, Chobanian & Avedisian School of Medicine, Boston University, Boston, Massachusetts; 6 Section of Infectious Diseases, Department of Medicine, Boston Medical Center and Chobanian & Avedisian School of Medicine, Boston University, Boston, Massachusetts; 7 Department of Public Health and Community Medicine, Tufts University School of Medicine, Boston, Massachusetts; 8 Division of Infectious Diseases, College of Medicine, University of Kentucky, Lexington; 9 Center on Drug and Alcohol Research, University of Kentucky, Lexington; 10 Department of Biostatistics, College of Public Health, University of Kentucky, Lexington; 11 Kentucky Injury Prevention Research Center, University of Kentucky, Lexington; 12 Department of Behavioral Science and Psychiatry, University of Kentucky, Lexington; 13 Division of Health Services Research and Policy, Department of Public Health Sciences, University of Miami Miller School of Medicine, Miami, Florida; 14 Department of Emergency Medicine, College of Medicine, The Ohio State University, Columbus; 15 Division of Community Health Sciences, School of Public Health, University of California, Berkeley

## Abstract

**Importance:**

Local-level data are needed to understand whether the relaxation of X-waiver training requirements for prescribing buprenorphine in April 2021 translated to increased buprenorphine treatment.

**Objective:**

To assess whether relaxation of X-waiver training requirements was associated with changes in the number of clinicians waivered to and who prescribe buprenorphine for opioid use disorder and the number of patients receiving treatment.

**Design, Setting, and Participants:**

This serial cross-sectional study uses an interrupted time series analysis of 2020-2022 data from the HEALing Communities Study (HCS), a cluster-randomized, wait-list–controlled trial. Urban and rural communities in 4 states (Kentucky, Massachusetts, New York, and Ohio) with a high burden of opioid overdoses that had not yet received the HCS intervention were included.

**Exposure:**

Relaxation of X-waiver training requirements (ie, allowing training-exempt X-waivers) on April 28, 2021.

**Main Outcomes and Measures:**

The monthly number of X-waivered clinicians, X-waivered buprenorphine prescribers, and patients receiving buprenorphine were each summed across communities within a state. Segmented linear regression models to estimate pre– and post–policy change by state were used.

**Results:**

The number of individuals in 33 participating HCS communities included 347 863 in Massachusetts, 815 794 in Kentucky, 971 490 in New York, and 1 623 958 in Ohio. The distribution of age (18-35 years: range, 29.4%-32.4%; 35-54 years: range, 29.9%-32.5%; ≥55 years: range, 35.7%-39.3%) and sex (female: range, 51.1%-52.6%) was similar across communities. There was a temporal increase in the number of X-waivered clinicians in the pre–policy change period in all states, which further increased in the post–policy change period in each state except Ohio, ranging from 5.2% (95% CI, 3.1%-7.3%) in Massachusetts communities to 8.4% (95% CI, 6.5%-10.3%) in Kentucky communities. Only communities in Kentucky showed an increase in the number of X-waivered clinicians prescribing buprenorphine associated with the policy change (relative increase, 3.2%; 95% CI, 1.5%-4.9%), while communities in other states showed no change or a decrease. Similarly, only communities in Massachusetts experienced an increase in patients receiving buprenorphine associated with the policy change (relative increase, 1.7%; 95% CI, 0.8%-2.6%), while communities in other states showed no change.

**Conclusions and Relevance:**

In this serial cross-sectional study, relaxation of X-waiver training requirements was associated with an increase in the number of X-waivered clinicians but was not consistently associated with an increase in the number of buprenorphine prescribers or patients receiving buprenorphine. These findings suggest that training requirements may not be the primary barrier to expanding buprenorphine treatment.

## Key Points

**Question:**

What is the association of the relaxation of training requirements to prescribe buprenorphine for opioid use disorder in April 2021 with changes in the number of clinicians eligible to and who prescribe buprenorphine and the number of patients receiving buprenorphine treatment?

**Findings:**

In this serial cross-sectional study of 33 communities from 4 states with high burdens of opioid overdose from May 2020 to May 2022, relaxing buprenorphine training requirements was associated with an increase in the number of clinicians eligible to prescribe buprenorphine. However, in general, no change in the number of clinicians prescribing buprenorphine or patients receiving buprenorphine treatment was found.

**Meaning:**

These findings suggest that training requirements may not be the primary barrier to increasing buprenorphine care, highlighting the need for ongoing multilevel interventions to expand buprenorphine treatment.

## Teaser

This cross-sectional study evaluates whether relaxation of X-waiver training requirements is associated with increases in buprenorphine treatment for patients with opioid use disorder.

## Introduction

Buprenorphine is a highly effective treatment for opioid use disorder (OUD) that prevents morbidity and mortality.^1^ However, expanding buprenorphine treatment access and uptake has been limited relative to the burden of opioid-related overdose deaths, with geographic, racial, and gender inequities observed.^2,3,4,5,6,7^ Historically, a frequently cited barrier has been the requirement that clinicians undergo special training in order to prescribe buprenorphine for OUD, commonly known as the X-waiver. Originating from the Drug Addiction Treatment Act of 2000, the X-waiver required additional training for prescribers licensed by the US Drug Enforcement Administration (DEA), including 8 hours for physicians and 24 hours for advanced practitioners.^8^ Critics of the X-waiver pointed out that the additional training, which is unique to buprenorphine, was onerous to clinicians and stigmatized both patients with OUD and the medication itself.^9,10,11,12^ Others have noted that the X-waiver may not be a primary barrier to treatment, as many X-waivered clinicians do not actively prescribe buprenorphine.^13,14^

In response to the ongoing rise in opioid overdose deaths, the X-waiver requirement was formally repealed as part of the Consolidated Appropriations Act on December 29, 2022.^15^ Prior to this, however, on April 28, 2021, the Biden Administration relaxed X-waiver training requirements by allowing clinicians to apply for a training exemption to prescribe buprenorphine for a maximum of 30 patients.^16^ Evaluating whether this earlier training exemption had the intended outcome of increasing the availability of buprenorphine is important to help guide expectations and additional efforts to expand buprenorphine treatment since the recent repeal of the X-waiver program.

Several studies have evaluated the outcomes of relaxing X-waiver training requirements in April 2021 and showed small increases in the total number of X-waivered clinicians, largely driven by those with new training-exempt 30-patient waivers.^17,18^ Other recent studies have evaluated national trends in buprenorphine prescriptions and clinicians prescribing buprenorphine without explicitly evaluating the April 2021 policy changes.^19,20^ These prior studies are limited by a sizeable proportion of clinicians with X-waivers not prescribing buprenorphine and the use of national data that may obscure state-level variation in responses to policy changes. Therefore, additional studies linking granular data on X-waivered clinicians with prescribing and claims data at a more local level are needed to investigate whether the relaxed training requirements have translated to more buprenorphine treatment in geographic areas of high treatment need.^21,22,23^ Using data from urban and rural communities in states with a high burden of opioid overdoses, we assessed whether relaxing the X-waiver training requirements would result in changes in (1) the number of X-waivered clinicians, (2) the number of X-waivered clinicians who prescribe buprenorphine, and (3) the number of patients receiving buprenorphine.

## Methods

This cross-sectional study was approved by Advarra Inc, the HEALing Communities Study (HCS) single institutional review board. The study was granted a waiver of consent and a full waiver of Health Insurance Portability and Accountability Act authorization for secondary data analysis. It was given a waiver of consent because it was impracticable for researchers to obtain written consent from all research participants. The study follows the Strengthening the Reporting of Observational Studies in Epidemiology (STROBE) reporting guideline.^24^

### Study Sample and Data Sources

We used data from the HCS, a 4-year, multisite, parallel-group, cluster-randomized, wait-list–controlled trial testing community-level interventions to reduce opioid overdose deaths in 67 communities located in 4 states (Kentucky, Massachusetts, New York, and Ohio). The HCS protocol has been described elsewhere.^25^ The HCS communities were selected based on a variety of criteria, including a high burden of opioid overdose deaths and a willingness to implement evidence-based interventions to reduce opioid overdoses (including expanding medications for OUD). Communities were randomized to active intervention (wave 1), which started interventions in year 1 of the study, or to the wait-list control (wave 2), which started interventions in year 3. We used monthly data collected as part of HCS from May 1, 2020, through May 31, 2022, from the 33 wave 2 communities that had not yet started a community-level intervention (eTable 1 in Supplement 1). We did not include wave 1 communities because community-level interventions may have increased the number of X-waivered clinicians and buprenorphine prescribing during the same period as the policy change. Data on the number of X-waivered clinicians in each community overall and by patient limit level (30, 100, and 275) were from the DEA files. The number of X-waivered clinicians prescribing buprenorphine was obtained by merging the DEA file data with state prescription drug monitoring program (PDMP) data that included all controlled substances dispensed in state pharmacies. The number of patients who were residents of study communities and prescribed buprenorphine were from PDMP data. Data on population size and sociodemographic characteristics of communities were from the US Census Bureau’s 2014-2018 American Community Survey 5-year estimates^26^ and the 2020 bridged race population estimates.^27^

### Study Exposure

The exposure of interest was change in the X-waiver training requirements. Though the requirements changed on April 28, 2021, due to software updates needed to accommodate the updated requirements, June 2021 was the earliest month that a clinician could receive training-exempt X-waiver status. Therefore, June 2021 was treated as the first month that the policy could affect outcomes.

According to the new guidelines, clinicians were able to submit a Notice of Intent application to treat up to 30 patients with buprenorphine without undergoing the X-waiver training.^16^ Despite the change in federal guidance, some states had preexisting state policies that mandated training and superseded the federal changes. In our sample, Kentucky administrative regulations continued to require all clinicians to obtain buprenorphine certification through completion of a Substance Abuse and Mental Health Services Administration–certified course.^28^ No other states in our sample continued to require further training to prescribe buprenorphine for OUD.

### Study Outcomes

The 3 primary outcomes included the total number of X-waivered clinicians, the number of X-waivered clinicians who prescribed buprenorphine to at least 1 patient, and the number of patients receiving buprenorphine. We chose to evaluate the total number of X-waivered clinicians rather than focus solely on those with 30-patient waivers for 2 reasons. First, the number of X-waivered clinicians in each patient waiver category is dynamic over time. Clinicians can transition from the 30-patient waiver category to higher patient waiver categories or drop out of higher patient waiver categories due to retirement or moving out of state. Focusing solely on clinicians with a 30-patient waiver may therefore give an incomplete picture of the policy’s association with buprenorphine treatment capacity. Second, the total number of X-waivered clinicians is ultimately of most importance for treatment access, and clinicians with higher patient limits are generally more likely to prescribe buprenorphine.^20^ We performed additional analyses using X-waivered clinicians with 30-patient waivers as our outcome (see the Sensitivity Analysis section). Ohio and Massachusetts suppressed data on the number of X-waivered clinicians prescribing buprenorphine in several communities due to counts less than 5. To account for these suppressed data, the outcome was estimated using multiple imputation. Details about suppressed data are provided in eTable 2 in Supplement 1.

### Statistical Analysis

#### Main Analysis

We described community sociodemographic characteristics, including age, sex, and race and ethnicity (Hispanic, non-Hispanic Black, non-Hispanic White, and non-Hispanic other [including American Indian or Alaska Native, Asian, Native Hawaiian or Pacific Islander, and other not otherwise specified]), and X-waivered clinician numbers in participating communities by state using counts and percentages for categorical variables and means and standard deviations for continuous variables. We then analyzed monthly outcomes divided into 2 periods: May 1, 2020, to May 31, 2021 (pre period), and June 1, 2021, to May 31, 2022 (post period). Totals for each outcome were calculated as the sum of the outcomes from each community within a state. To evaluate the association of relaxing the X-waiver training requirement with each outcome, we used state-level interrupted time series (ITS) models. These segmented linear regression models included terms for baseline level and time trend in the pre period as well as change in level and time trend in the post period to examine immediate effects and changes to the slope, respectively. Because implementation of the federal guidance varied by state, models were run separately by state. Given that small counts for number of X-waivered clinicians prescribing buprenorphine were suppressed in Ohio and Massachusetts, we used multiple imputation to include all communities in the analysis. To improve the power to detect outcome changes associated with the policy and avoid inclusion of terms that might obscure trends, we sequentially removed terms from the ITS models with *P* > .20, except for the preperiod time trend. We used a stepwise autoregression approach to test and control for autocorrelation starting with 12 lags and using the exact maximum likelihood method to select a final model with appropriate autoregressive terms.^29,30^ We used regression results to estimate absolute and relative changes in the outcomes at 12 months after the policy change using multivariable delta methods.^31^ We used SAS, version 9.4 (SAS Institute Inc) for all analyses, and all hypothesis tests were 2-sided with an a priori level of significance of *P* < .05.

#### Sensitivity Analysis

Because the X-waiver training relaxation only allowed clinicians to enter the 30-patient waiver category, we reran our segmented regression models using the number of X-waivered clinicians with 30-patient waivers as the outcome. To assess the sensitivity of our imputation methods for suppressed data on the number of X-waivered clinicians prescribing buprenorphine in several communities in Ohio and Massachusetts, we reran models excluding the observations with suppressed data. To evaluate whether the policy change resulted in changes in the geographic availability of X-waivered clinicians, we also calculated the number of zip codes in study communities with X-waivered clinicians in the pre and post periods, including the number of zip codes where a new training-exempt X-waivered clinician was the only X-waivered clinician.

## Results

### Study Sample

Table 1 summarizes the characteristics of 33 study communities by state, which included populations of 347 863 individuals in Massachusetts, 815 794 in Kentucky, 971 490 in New York, and 1 623 958 in Ohio. The distribution of age (18-35 years: range, 29.4%-32.4%; 35-54 years: range, 29.9%-32.5%; ≥55 years: range, 35.7%-39.3%) and sex (female: range, 51.1%-52.6%; male: range, 47.4%-48.9%) was similar across communities in HCS states. The typical study community in all states was majority non-Hispanic White (range, 59.4%-76.4%), with variable proportions of Hispanic (range, 3.8%-28.3%), non-Hispanic Black (range, 7.7%-16.6%), and non-Hispanic other (2.8%-4.6%) residents. The number of X-waivered clinicians and their distribution by patient limits varied considerably across states. Clinicians with 30-patient limits made up the majority of X-waivered clinicians in communities in each state both before and after the policy change (range, 57.4%-72.8%). A minority of clinicians with X-waivers actively prescribed buprenorphine before and after the policy change (range, 26.6%-44.7%) (Table 1). Compared with May 2020, the proportion of X-waivered clinicians with a 30-patient limit who actively prescribed buprenorphine decreased after the policy change in all states but Ohio. eTable 3 in Supplement 1 shows the percentage change in each X-waivered clinician category comparing May 2020 and May 2022.

**Table 1.** Characteristics of HEALing Communities Study Sites by State^a^

Baseline characteristic^b^	No. of individuals (%)			
	Kentucky	Massachusetts	New York	Ohio
**Community population**				
No. of communities	8	8	8	9
Total population	815 764	347 863	971 490	1 623 958
Age group, y				
18-34	239 713 (29.4)	108 148 (31.1)	300 494 (30.9)	526 187 (32.4)
35-54	255 345 (31.3)	113 144 (32.5)	290 916 (29.9)	518 276 (31.9)
≥55	320 706 (39.3)	126 571 (36.4)	380 080 (39.1)	579 495 (35.7)
Race and ethnicity				
Hispanic	35 547 (4.4)	98 429 (28.3)	115 287 (11.9)	62 397 (3.8)
Non-Hispanic Black	134 180 (16.4)	26 805 (7.7)	115 154 (11.9)	270 005 (16.6)
Non-Hispanic White	623 388 (76.4)	206 657 (59.4)	701 153 (72.2)	1 218 655 (75.0)
Non-Hispanic other^c^	22 649 (2.8)	15 972 (4.6)	39 896 (4.1)	72 901 (4.5)
Sex				
Female	426 312 (52.3)	183 125 (52.6)	496 734 (51.1)	836 526 (51.5)
Male	389 452 (47.7)	164 738 (47.4)	474 756 (48.9)	787 432 (48.5)
Median community income, mean (SD), $	25 127.9 (6582.4)	31 953.5 (8358.7)	27 426.1 (3870.1)	27 620.1 (3119.6)
Prepolicy (May 2020) monthly opioid overdose deaths per 100 000 adult residents, mean (SD)	7.0 (4.2)	7.9 (6.8)	4.2 (3.0)	6.9 (5.9)
Prepolicy (May 2020) monthly nonfatal opioid overdose events per 100 000 adult residents, mean (SD)	36.2 (15.1)	36.0 (18.9)	13.4 (4.7)	23.4 (4.1)
**X-waivered clinicians by patient limits** ^d^				
Prepolicy (May 2020)	317	589	528	1027
30 Patients	182 (57.4)	429 (72.8)	369 (69.9)	729 (71.0)
100 Patients	88 (27.8)	116 (19.7)	108 (20.5)	181 (17.6)
275 Patients	47 (14.8)	44 (7.5)	51 (9.7)	117 (11.4)
Postpolicy (May 2022)	439	738	724	1362
30 Patients, exempt	61 (13.9)	68 (9.2)	79 (10.9)	131 (9.6)
30 Patients, total	258 (58.8)	506 (68.6)	487 (67.3)	921 (67.6)
100 Patients	109 (24.8)	174 (23.6)	169 (23.3)	282 (20.7)
275 Patients	72 (16.4)	58 (7.9)	68 (9.4)	159 (11.7)
**X-waivered clinicians who prescribed buprenorphine to community residents by patient limits** ^e^ ^,^ ^f^				
Prepolicy (May 2020)	120 (37.9)	165 (28.0)	236 (44.7)	314 (30.6)
30 Patients	30 (16.5)	84 (19.6)	112 (30.4)	94 (12.9)
100 Patients	48 (54.5)	50 (43.1)	78 (72.2)	101 (55.8)
275 Patients	42 (89.4)	18 (40.9)	46 (90.2)	85 (72.6)
Postpolicy (May 2022)	125 (28.5)	196 (26.6)	295 (40.7)	398 (29.2)
30 Patients	29 (11.2)	78 (15.4)	129 (26.5)	139 (15.1)
100 Patients	42 (38.5)	78 (44.8)	105 (62.1)	140 (49.6)
275 Patients	54 (75.0)	40 (69.0)	61 (89.7)	119 (74.8)

^a^ Communities were those in the wait-list control arm (wave 2) of the randomized clinical trial and, thus, did not receive any intervention throughout the study period. All values correspond to the HEALing Communities Study baseline in 2019 unless otherwise noted.

^b^ Percentages may not add up to 100 due to rounding. For communities that represent counties (24 of 33), population information was drawn from 2020 US Census Bureau bridged race population estimates.^27^

^c^ Other race and ethnicity included American Indian or Alaska Native, Asian, Native Hawaiian or Pacific Islander, and other not otherwise specified.

^d^ Of total X-waivered clinicians.

^e^ Includes only X-waivered clinicians who prescribe buprenorphine products that are US Food and Drug Administration approved for opioid use disorder to at least 1 patient. Due to the suppression of small counts, the sum of values for subgroups by patient count may not match the overall values. For communities that represent units smaller than counties (9 of 33), population information was drawn from the 2014-2018 American Community Survey 5-year estimates.^26^

^f^ Of total X-waivered clinicians by category.

### Number of X-Waivered Clinicians

**Figure 1.**
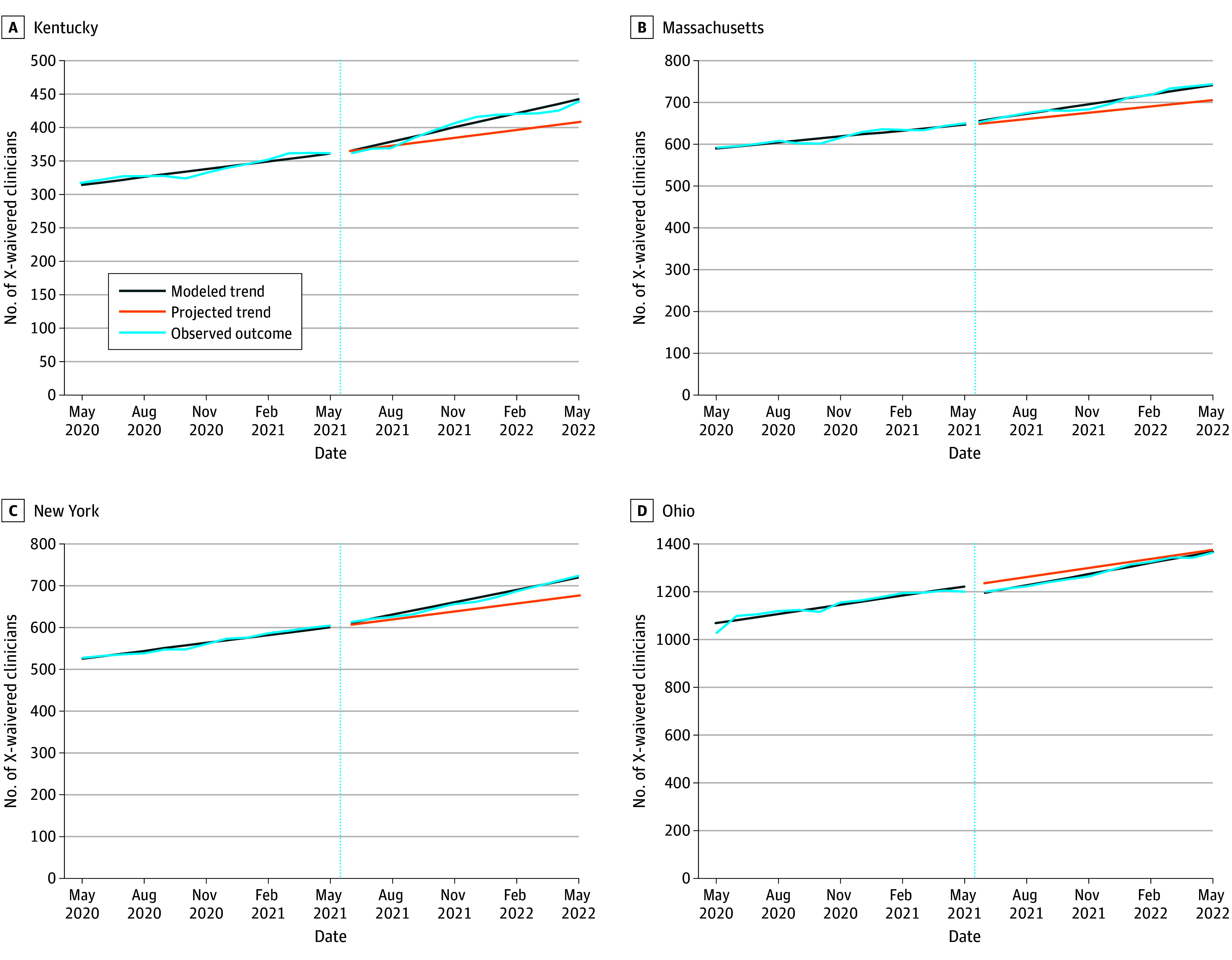
Number of X-Waivered Clinicians Before and After Relaxation of the X-Waiver Training Requirements, May 2020 to May 2022 Dotted vertical lines indicate the time of the X-waiver training relaxation. Projected trends are based on preperiod data. Modeled trend lines show the outcome accounting for level and linear trend changes due to the X-waiver training relaxation.

The total number of X-waivered clinicians increased over time, with communities in 3 of 4 states showing a greater rate of increase after the X-waiver training relaxation took effect in June 2021 (Table 2; Figure 1). For instance, in communities in Kentucky, Massachusetts, and New York, temporal increases in X-waivered clinicians grew by an additional 3.1 (95% CI, 2.6-3.7), 3.0 (95% CI, 1.8-4.2), and 3.6 (95% CI, 1.8-5.4) clinicians per month after the X-waiver training was relaxed, respectively. Communities in Ohio had a decrease in the number of X-waivered clinicians immediately after the policy change (41.5 fewer clinicians in June 2021 compared with May 2021; 95% CI, 20.5-62.5 fewer clinicians) but greater temporal growth in X-waivered clinicians in the post period (an additional 2.9 clinicians per month compared with the pre period; 95% CI, −0.0 to 5.8 additional clinicians). By May 2022, the policy change was associated with increases in the number of X-waivered clinicians compared with the expected number based on baseline trends in communities in all states but Ohio, ranging from 5.2% (95% CI, 3.1%-7.3%) in Massachusetts (absolute change, 36.3 [95% CI, 22.0-50.6] additional clinicians) to 8.4% (95% CI, 6.5%-10.3%) in Kentucky (absolute change, 34.3 [95% CI, 27.1-41.6] additional clinicians) (Table 2).

**Table 2.** Estimated Changes in Each Outcome Associated With X-Waiver Training Relaxation, May 2020 to May 2022

Outcome by state	Model variable (95% CI)^a^				Estimated change in outcomes 12 mo after policy change (95% CI)^b^	
	Intercept	Preperiod linear trend	Postperiod level change	Postperiod linear trend change	Absolute, No.	Relative, %
**No. of X-waivered clinicians**						
Kentucky	309.9 (306.9 to 312.9)	3.9 (3.5 to 4.3)	−3.0 (−7.3 to 1.4)	3.1 (2.6 to 3.7)	34.3 (27.1 to 41.6)	8.4 (6.5 to 10.3)
Massachusetts	581.1 (575.1 to 587.0)	4.8 (4.1 to 5.4)	NA	3.0 (1.8 to 4.2)	36.3 (22.0 to 50.6)	5.2 (3.1 to 7.3)
New York	519.0 (510.1 to 527.9)	6.3 (5.3 to 7.3)	NA	3.6 (1.8 to 5.4)	43.4 (22.7 to 64.1)	6.4 (3.2 to 9.6)
Ohio	1056.0 (1040.3 to 1070.9)	12.7 (10.8 to 14.6)	−41.5 (−62.5 to −20.5)	2.9 (−0.0 to 5.8)	−6.8 (−45.0 to 31.4)	−0.5 (−3.3 to 2.3)
**No. of X-waivered clinicians prescribing buprenorphine to ≥1 patient^c^**						
Kentucky	120.9 (118.8 to 123.0)	−0.2 (−0.5 to 0.1)	3.6 (−0.0 to 7.1)	NA	3.7 (1.8 to 5.6)	3.2 (1.5 to 4.9)
Massachusetts	173.5 (165.7 to 181.3)	1.5 (0.5 to 2.4)	13.1 (2.3 to 23.8)	−1.8 (−3.2 to −0.3)	−8.0 (−27.4 to 11.5)	−3.8 (−12.8 to 5.2)
New York	231.2 (227.6 to 234.8)	2.3 (2.1 to 2.6)	NA	NA	NA	NA
Ohio	326.2 (315.6 to 336.8)	3.7 (2.5 to 4.8)	NA	−2.4 (−4.5 to −0.3)	−28.8 (−54.0 to −3.5)	−6.9 (−12.6 to −1.2)
**No. of patients receiving buprenorphine**						
Kentucky	5892.0 (5803.3 to 5981.4)	40.0 (28.8 to 51.2)	96.3 (−26.1 to 218.7)	−16.7 (−33.7 to 0.2)	−104.7 (−326.7 to 117.3)	−1.5 (−4.7 to 1.7)
Massachusetts	4262.0 (4247.4 to 4276.0)	−12.1 (−13.9 to −10.3)	−19.3 (−39.8 to 1.2)	7.1 (4.5 to 9.7)	66.0 (31.6 to 100.5)	1.7 (0.8 to 2.6)
New York	6545.0 (6481.9 to 6608.4)	2.2 (−2.0 to 6.5)	NA	NA	NA	NA
Ohio	10 254.0 (10 137.2 to 10 370.5)	36.1 (21.4 to 50.8)	151.9 (−8.4 to 312.2)	−34.0 (−56.2 to −11.9)	−256.2 (−547.1 to 34.6)	−2.3 (−4.9 to 0.3)

Abbreviation: NA, not applicable.

^a^ Estimates from interrupted time series (ITS) models. All models used stepwise autoregression to control for serial autocorrelation. Not applicable indicates that a postperiod-level change or postperiod linear trend change was no different from the preperiod trend at the *P* < .20 level. The intercept term is the mean outcome value in May 2020. The preperiod linear trend is the mean monthly change in the outcome in the pre period. The postperiod-level change is the instantaneous mean change in the outcome in the month immediately following the policy change. The postperiod linear trend change is the mean monthly change in the outcome in the post period compared with what the mean monthly change would have been had the preperiod trend continued.

^b^ Absolute change calculated as the difference between ITS models with only intercept and preperiod time trend vs ITS models with intercept, preperiod time trend, and either or both the postperiod-level change and slope change terms as shown in the table. The 95% CIs were calculated using the delta method. Not applicable indicates that a change could not be calculated because the policy change was not associated with a postperiod-level or slope change in the ITS models at the *P* < .20 level.

^c^ Massachusetts and Ohio models include imputed data for community-months with suppressed data. eTable 2 in Supplement 1 provides additional details.

### Number of X-Waivered Clinicians Prescribing Buprenorphine

**Figure 2.**
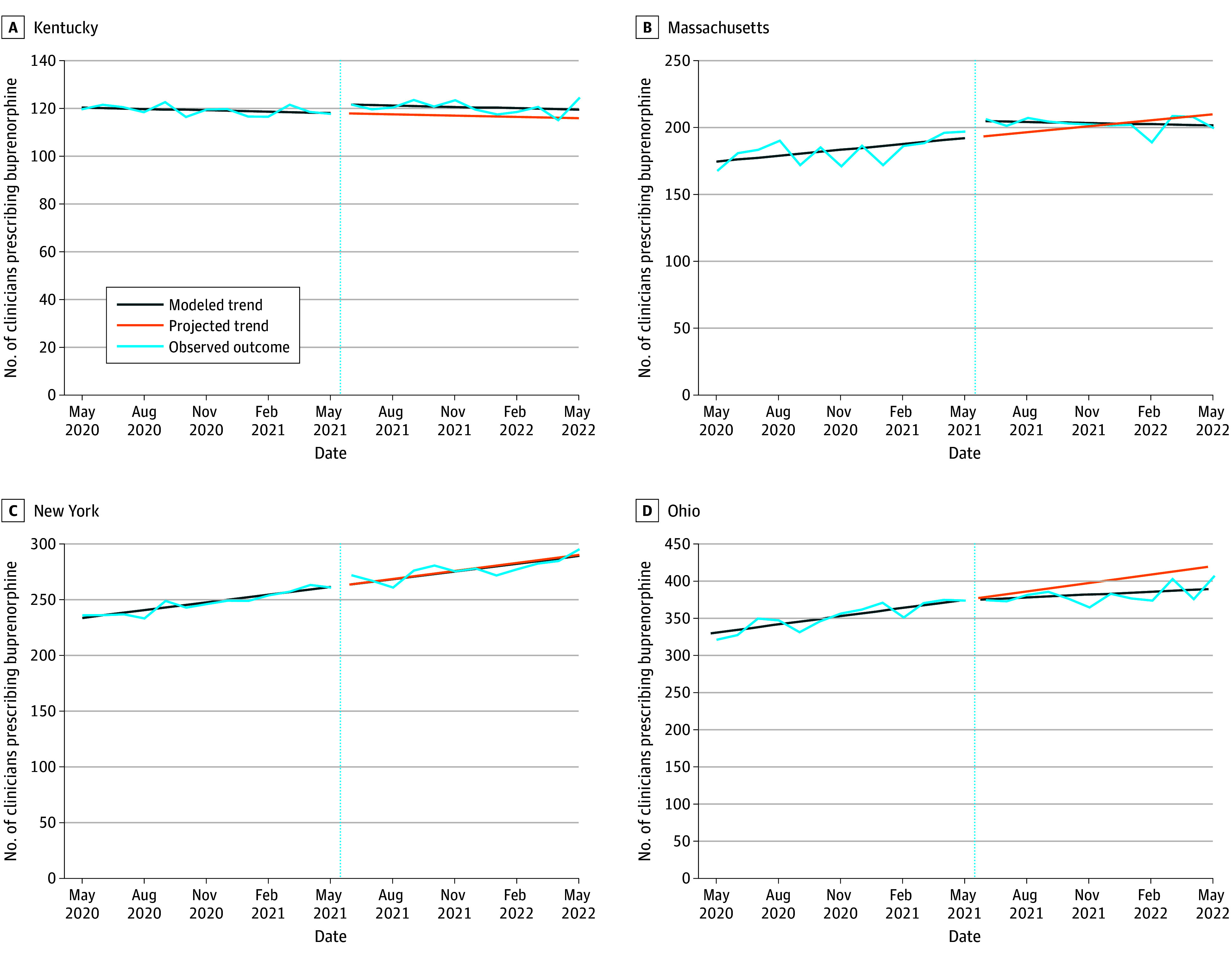
Number of X-Waivered Clinicians Prescribing Buprenorphine Before and After Relaxation of the X-Waiver Training Requirements, May 2020 to May 2022 Dotted vertical lines indicate the time of the X-waiver training relaxation. Projected trends are based on preperiod data. Modeled trend lines show the outcome accounting for level and linear trend changes due to the X-waiver training relaxation.

Prior to the policy change, the number of X-waivered clinicians prescribing buprenorphine was increasing in communities in Massachusetts, New York, and Ohio, but not Kentucky (Table 2; Figure 2). After X-waiver training was relaxed, communities in Kentucky and Massachusetts experienced immediate increases in the number of X-waivered clinicians prescribing buprenorphine (3.6 [95% CI, −0.0 to 7.1] and 13.1 [95% CI, 2.3-23.8] additional clinicians, respectively). However, the temporal trend in the number of X-waivered clinicians prescribing buprenorphine did not increase in communities in any state and decreased in communities in Massachusetts and Ohio by 1.8 (95% CI, 0.3-3.2) and 2.4 (95% CI, 0.3-4.5) clinicians per month, respectively. By May 2022, the policy change was associated with no overall change in the number of X-waivered clinicians prescribing buprenorphine in communities in Massachusetts and New York, a 3.2% (95% CI, 1.5%-4.9%) increase in Kentucky communities, and a 6.9% (95% CI, 1.2%-12.6%) decrease in Ohio communities (Table 2).

### Number of Patients Receiving Buprenorphine

**Figure 3.**
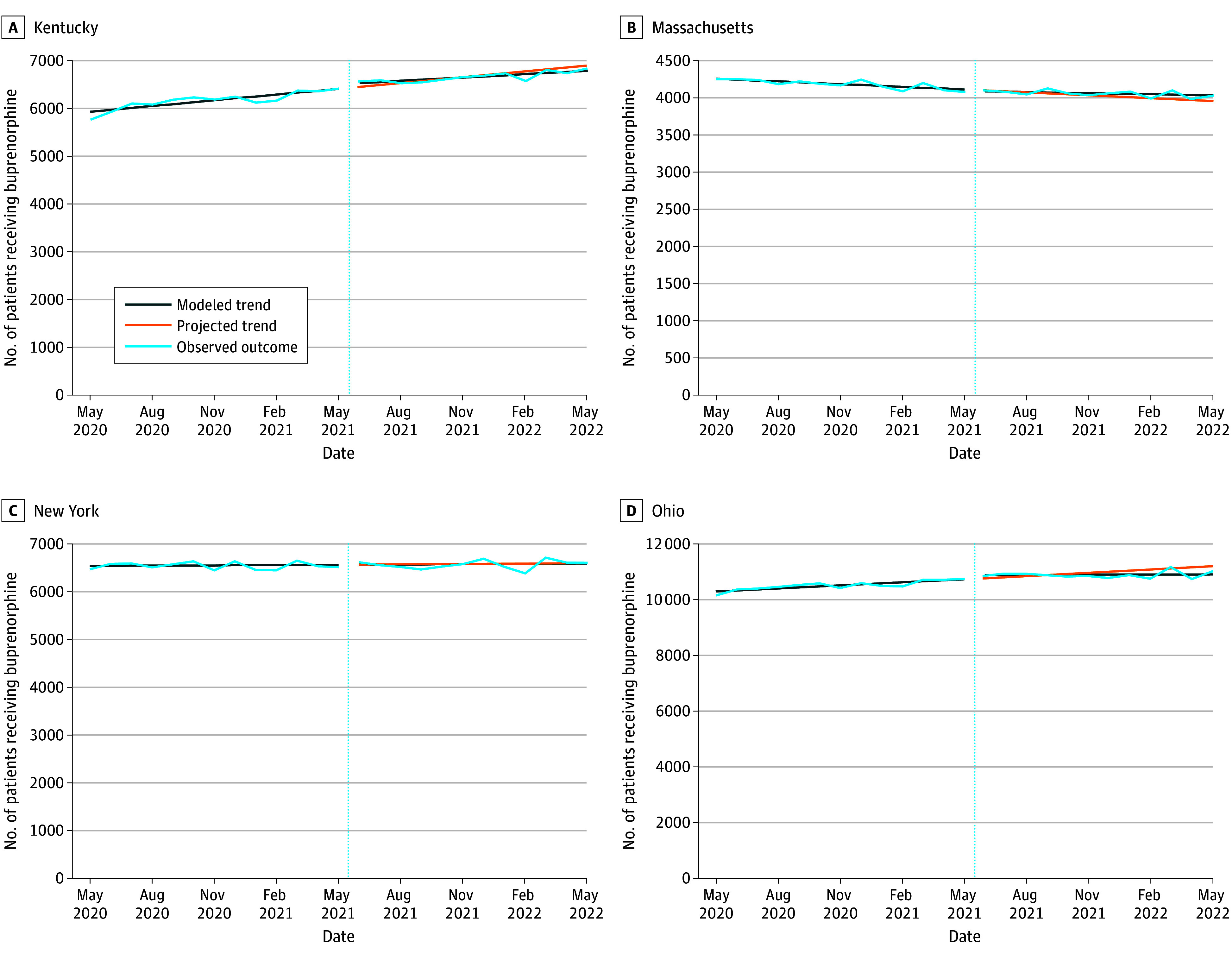
Number of Patients Receiving Buprenorphine Before and After Relaxation of the X-Waiver Training Requirements, May 2020 to May 2022 Dotted vertical lines indicate the time of the X-waiver training relaxation. Projected trends are based on preperiod data. Modeled trend lines show the outcome accounting for level and linear trend changes due to the X-waiver training relaxation.

The number of patients prescribed buprenorphine was increasing prior to the policy change for communities in Kentucky and Ohio but not Massachusetts or New York (Table 2; Figure 3). After the X-waiver training requirement was relaxed, no state experienced an immediate change in patients receiving buprenorphine. The post period changes in temporal trends showed a slowing of the number of patients receiving buprenorphine in communities in Ohio (34.0 [95% CI, 11.9-56.2] fewer patients per month). Only communities in Massachusetts showed a positive change in the postperiod temporal trend of patients prescribed buprenorphine (7.1 additional patients per month compared with the preperiod trend; 95% CI, 4.5 to 9.7 additional patients). By May 2022, X-waiver training relaxation was associated with a 1.7% (95% CI, 0.8%-2.6%) increase in patients receiving buprenorphine in communities in Massachusetts compared with projected trends (absolute change, 66.0 [95% CI, 31.6-100.5] additional patients) but was associated with no change in communities in all other states (Table 2).

### Sensitivity Analysis

Regression models using the number of X-waivered clinicians with 30-patient waivers as the outcome showed that relaxing X-waiver training was associated with increases in clinicians with 30-patient waivers (eTable 4; eFigure in Supplement 1). By May 2022, the number of clinicians with 30-patient waivers exceeded projected trends in communities in all states, ranging from 5.3% (95% CI, 1.0%-10.0%) in New York and 5.3% (95% CI, 2.8%-7.9%) in Ohio to 24.6% (95% CI, 20.2%-29.1%) in Kentucky communities (eTable 4 in Supplement 1). Regression models excluding suppressed data for the number of X-waivered clinicians prescribing buprenorphine in communities in Ohio and Massachusetts resulted in similar effect estimates as models using imputed data (eTable 5 in Supplement 1). The geographic distribution of X-waivered clinicians was largely unchanged after the policy change (eTable 6 in Supplement 1). Only 2 zip codes had no prior X-waivered clinicians and gained access via new training-exempt X-waivered clinicians.

## Discussion

In this serial cross-sectional study of 33 communities from 4 states with high burdens of opioid-related morbidity and mortality, relaxing X-waiver training requirements had mixed associations with buprenorphine treatment. The X-waiver training relaxation was associated with faster growth in the number of X-waivered clinicians in communities in 3 of the 4 states. However, the policy change was generally not associated with increases in the number of X-waiver clinicians prescribing buprenorphine or the number of patients receiving buprenorphine. Overall, these findings suggest that while relaxing X-waiver training requirements may be important to increasing the supply of potential clinicians, by itself, it may not lead to increases in the number of patients receiving buprenorphine.

Initially hailed as progressive for its effort to expand evidence-based OUD treatment into general medical settings, the Drug Addiction Treatment Act of 2000 X-waiver requirement was increasingly cited as a barrier to care. Critics of the X-waiver pointed out that the additional training was onerous and stigmatized both patients with OUD and the medication itself.^10^ By requiring additional training, the X-waiver singled out buprenorphine as uniquely dangerous despite its favorable safety profile.^11,32^ Ongoing increases in fatal opioid overdoses during the COVID-19 pandemic combined with advocacy efforts led to the relaxation of X-waiver training requirements in April 2021 and ultimately the repeal of the X-waiver in December 2022.^33^

While support for removing the X-waiver was broad, how it might affect buprenorphine treatment is debated. Some hypothesized that simply removing the X-waiver may not lead to a substantial increase in buprenorphine prescribing due to the multiple other systems issues affecting buprenorphine care, including DEA audits, state requirements, inadequate clinical support, and insurance requirements such as prior authorizations.^13,34,35^ Our findings suggest that the removal of X-waiver training requirements by itself may not lead to substantial increases in the number of clinicians prescribing buprenorphine or the number of patients receiving buprenorphine treatment in the short term. This observation is consistent with findings that many clinicians who obtain X-waivers do not prescribe buprenorphine or only prescribe to a small number of patients.^14,36,37^

### Limitations

This study has several limitations. First, the study population was limited to a selection of communities in 4 states with a high burden of opioid-related morbidity and mortality. While this study population allowed us to evaluate multiple outcomes relevant to the buprenorphine treatment process, whether the patterns observed in these communities are representative of trends throughout the US remains unknown. Second, due to the relatively small number of communities included in each state, our results may be more sensitive to the opening or closing of high-volume outpatient buprenorphine clinics. Third, our data cover a short period before and after the X-waiver training relaxation was implemented, and it is possible that the outcomes of the policy change may continue to evolve over time. Fourth, we do not have data on methadone treatment. Contemporaneous COVID-related methadone regulatory changes, along with challenges to initiating buprenorphine due to fentanyl penetration of the opioid supply, may have made methadone more appealing to some patients.^38,39,40^ Fifth, using PDMP data for buprenorphine prescriptions may capture some patients receiving buprenorphine for pain rather than OUD and omit buprenorphine administered in treatment facilities rather than prescribed. However, neither of these limitations would be expected to change contemporaneously with the policy under study. Finally, relaxing X-waiver training requirements, which still required clinicians to fill out an X-waiver exemption form, is different from removing the X-waiver, as took place in December 2022. Whether completely removing the X-waiver will have different outcomes remains to be seen, though our findings suggest that increases in buprenorphine prescribing observed with this policy change may be less pronounced than hoped for.

## Conclusions

The findings of this serial cross-sectional study of 4 states with a high burden of opioid-related morbidity and mortality show that relaxation of X-waiver training requirements in April 2021 was generally associated with an increase in the number of X-waivered clinicians but no consistent changes in the number of clinicians prescribing buprenorphine or the number of patients receiving buprenorphine for OUD. These findings support the ongoing need for multilevel interventions to expand buprenorphine treatment.^41^

## Notes

Supplement 1. **eTable 1.** List of Communities Included in Study Sample **eTable 2.** Suppressed Data on Number of X-Waivered Clinicians Who Actively Prescribe Buprenorphine Products That Are FDA Approved for OUD to HEALing Communities Study Residents in Ohio and Massachusetts, May 2020 to May 2022 **eTable 3.** Number and Percent Change of Clinicians Who Are X-Waivered and Who Prescribed Buprenorphine by Patient Limit Level, May 2020 to May 2022 **eTable 4.** Estimated Change in Number of X-Waivered Clinicians With a 30-Patient Limit Associated With X-Waiver Training Relaxation, May 2020 to May 2022 **eFigure.** Number of X-Waivered Clinicians With a 30-Patient Limit Before and After Relaxation of the X-Waiver Training Requirements, May 2020 to May 2022 **eTable 5.** Interrupted Time Series Sensitivity Analysis for the Effect of the X-Waiver Training Relaxation on the Number of X-Waivered Clinicians Prescribing Buprenorphine in Ohio and Massachusetts, Excluding Community-Months With Suppressed Data **eTable 6.** Number of Zip Codes With X-Waivered Clinicians in May 2021 and May 2022 and Training-Exempt X-Waivered Clinicians in May 2022

Supplement 2.
Data Sharing Statement
