# Supplementary material for: Buprenorphine Prescribing Characteristics Following Relaxation of X-Waiver Training Requirements
Source: JAMA Netw Open. 2024 Aug 5;7(8):e2425999. doi: 10.1001/jamanetworkopen.2024.25999 (PMC11301557; doi:10.1001/jamanetworkopen.2024.25999)
Supplement: Supplement 1. — eTable 1. List of Communities Included in Study Sample eTable 2. Suppressed Data on Number of X-Waivered Clinicians Who Actively Prescribe Buprenorphine Products That Are FDA Approved for OUD to HEALing Communities Study Residents in Ohio and Massachusetts, May 2020 to May 2022 eTable 3. Number and Percent Change of Clinicians Who Are X-Waivered and Who Prescribed Buprenorphine by Patient Limit Level, May 2020 to May 2022 eTable 4. Estimated Change in Number of X-Waivered Clinicians With a 30-Patient Limit Associated With X-Waiver Training Relaxation, May 2020 to May 2022 eFigure. Number of X-Waivered Clinicians With a 30-Patient Limit Before and After Relaxation of the X-Waiver Training Requirements, May 2020 to May 2022 eTable 5. Interrupted Time Series Sensitivity Analysis for the Effect of the X-Waiver Training Relaxation on the Number of X-Waivered Clinicians Prescribing Buprenorphine in Ohio and Massachusetts, Excluding Community-Months With Suppressed Data eTable 6. Number of Zip Codes With X-Waivered Clinicians in May 2021 and May 2022 and Training-Exempt X-Waivered Clinicians in May 2022 [file jamanetwopen-e2425999-s001.pdf]

Supplemental Online Content

Christine PJ, Chahine RA, Kimmel SD, et al. Buprenorphine prescribing characteristics following relaxation of X-waiver training requirements. *JAMA Netw Open*. 2024;7(8):e2425999. doi:10.1001/jamanetworkopen.2024.25999

eTable 1. List of Communities Included in Study Sample

eTable 2. Suppressed Data on Number of X-waivered Clinicians Who Actively Prescribe Buprenorphine Products That Are FDA Approved for OUD to HEALing Communities Study Residents in Ohio and Massachusetts, May 2020 to May 2022

eTable 3. Number and Percent Change of Clinicians Who Are X-Waivered and Who Prescribed Buprenorphine by Patient Limit Level, May 2020 to May 2022

eTable 4. Estimated Change in Number of X-Waivered Clinicians With a 30-Patient Limit Associated With X-Waiver Training Relaxation, May 2020 to May 2022

eFigure. Number of X-Waivered Clinicians With a 30-Patient Limit Before and After Relaxation of the X-Waiver Training Requirements, May 2020 to May 2022

eTable 5. Interrupted Time Series Sensitivity Analysis for the Effect of the X-Waiver Training Relaxation on the Number of X-Waivered Clinicians Prescribing Buprenorphine in Ohio and Massachusetts, Excluding Community-Months With Suppressed Data

eTable 6. Number of Zip Codes With X-Waivered Clinicians in May 2021 and May 2022 and Training-Exempt X-Waivered Clinicians in May 2022

This supplemental material has been provided by the authors to give readers additional information about their work.

© 2024 Christine PJ et al. *JAMA Network Open.*

eTable 1. List of Communities Included in Study Sample

State	Community Name	Rural/Urban
KY	Bourbon	Urban
KY	Campbell	Urban
KY	Carter	Rural
KY	Greenup	Urban
KY	Jefferson	Urban
KY	Jessamine	Urban
KY	Knox	Rural
KY	Mason	Rural
MA	North Adams	Urban
MA	Lawrence	Urban
MA	Springfield	Urban
MA	Pittsfield	Urban
MA	Weymouth	Urban
MA	Bristol (Berkeley/Dighton/Freetown)	Rural
MA	Franklin (Greenfield/Montague/Athol/Orange)	Rural
MA	Hampshire (Belchertown/Ware)	Rural
NY	Broome	Urban
NY	Chautauqua	Rural
NY	Cortland	Rural
NY	Genesee	Rural
NY	Monroe	Urban
NY	Orange	Urban
NY	Sullivan	Rural
NY	Yates	Urban
OH	Allen	Urban
OH	Brown	Urban
OH	Franklin	Urban
OH	Huron	Rural
OH	Jefferson	Urban
OH	Ross	Rural
OH	Stark	Urban
OH	Williams	Rural
OH	Wyandot	Rural

© 2024 Christine PJ et al. *JAMA Network Open.*

eTable 2. Suppressed Data on Number of X-Waivered Clinicians Who Actively Prescribe Buprenorphine Products That Are FDA Approved for OUD to HEALing Communities Study Residents in Ohio and Massachusetts, May 2020 to May 2022[a]

**Ohio**			**Massachusetts**		
**Community**	**Year**	**Number of Months With Suppressed Data, N (%)**	**Community**	**Year**	**Number of Months With Suppressed Data, N (%)**
Allen	2020	0 (0%)	Bristol (Berkley/Dighton/Freetown)	2020	0 (0%)
Allen	2021	0 (0%)	Bristol (Berkley/Dighton/Freetown)	2021	0 (0%)
Allen	2022	0 (0%)	Bristol (Berkley/Dighton/Freetown)	2022	4 (80%)
Brown	2020	6 (75%)	Franklin (Greenfield/Montague/Athol/ Orange)	2020	0 (0%)
Brown	2021	3 (25%)	Franklin (Greenfield/Montague/Athol/ Orange)	2021	0 (0%)
Brown	2022	5 (100%)	Franklin (Greenfield/Montague/Athol/ Orange)	2022	0 (0%)
Franklin	2020	0 (0%)	Hampshire (Belchertown/Ware)	2020	8 (100%)
Franklin	2021	0 (0%)	Hampshire (Belchertown/Ware)	2021	11 (92%)
Franklin	2022	0 (0%)	Hampshire (Belchertown/Ware)	2022	5 (100%)
Huron	2020	2 (25%)	Lawrence	2020	0 (0%)
Huron	2021	12 (100%)	Lawrence	2021	0 (0%)
Huron	2022	5 (100%)	Lawrence	2022	0 (0%)
Jefferson	2020	8 (100%)	North Adams	2020	8 (100%)
Jefferson	2021	12 (100%)	North Adams	2021	11 (92%)
Jefferson	2022	5 (100%)	North Adams	2022	1 (20%)
Ross	2020	0 (0%)	Pittsfield	2020	0 (0%)
Ross	2021	0 (0%)	Pittsfield	2021	0 (0%)
Ross	2022	0 (0%)	Pittsfield	2022	0 (0%)
Stark	2020	0 (0%)	Springfield	2020	0 (0%)
Stark	2021	0 (0%)	Springfield	2021	0 (0%)
Stark	2022	0 (0%)	Springfield	2022	0 (0%)
Williams	2020	8 (100%)	Weymouth	2020	0 (0%)
Williams	2021	12 (100%)	Weymouth	2021	0 (0%)
Williams	2022	5 (100%)	Weymouth	2022	0 (0%)
Wyandot	2020	3 (38%)			
Wyandot	2021	0 (0%)			
Wyandot	2022	0 (0%)			

[a] Data were suppressed when number of clinicians was less than five.

© 2024 Christine PJ et al. *JAMA Network Open.*

eTable 3. Number and Percent Change of Clinicians Who Are X-Waivered and Who Prescribed Buprenorphine by Patient Limit Level, May 2020 to May 2022[a]

	Kentucky			Massachusetts			New York			Ohio		
	Pre-Policy May 2020, n	Post-Policy May 2022, n	Percent Change, %	Pre-Policy May 2020, n	Post-Policy May 2022, n	Percent Change, %	Pre-Policy May 2020, n	Post-Policy May 2022, n	Percent Change, %	Pre-Policy May 2020, n	Post-Policy May 2022, n	Percent Change, %
**X-waivered clinicians by patient limits**												
Overall	317	439	38.5	589	738	25.3	528	724	37.1	1027	1362	32.6
30 patients	182	258	41.8	429	506	17.9	369	487	32.0	729	921	26.3
100 patients	88	109	23.9	116	174	50.0	108	169	56.5	181	282	55.8
275 patients	47	72	53.2	44	58	31.8	51	68	33.3	117	159	35.9
**X-waivered clinicians who prescribed buprenorphine to community residents by patient limits**												
Overall	120	125	4.2	165	196	18.8	236	295	25.0	314	398	26.8
30 patients	30	29	-3.3	84	78	-7.1	112	129	15.2	94	139	47.9
100 patients	48	42	-12.5	50	78	56.0	78	105	34.6	101	140	38.6
275 patients	42	54	28.6	18	40	122.2	46	61	32.6	85	119	40.0

[a] Due to the suppression of small counts, the sum of values for sub-groups by patient count may not match the overall values in Ohio and Massachusetts.

eTable 4. Estimated Change in Number of X-Waivered Clinicians With a 30-Patient Limit Associated With X-Waiver Training Relaxation, May 2020 to May 2022[a]

Outcome	Model Variable and 95% CI				Estimated change in outcomes 12 months after policy change (95% CI)[b]	
	Intercept	Pre-Period Linear Trend	Post-Period Level Change	Post-Period Linear Trend Change	Absolute Change	Relative Change, %
**Number of X-waivered clinicians with 30-patient waiver**						
Kentucky	176.9 (173.6 to 180.1)	1.3 (0.9 to 1.7)	NA	4.3 (3.6 to 5.0)	51.7 (43.9 to 59.5)	24.6 (20.2 to 29.1)
Massachusetts	423.7 (420.6 to 426.8)	1.5 (1.1 to 1.9)	-6.2 (-10.3 to -2.1)	4.4 (3.7 to 5.1)	46.9 (39.2 to 54.7)	10.2 (8.4 to 12.0)
New York	362.3 (357.6 to 367.0)	3.9 (3.3 to 4.4)	NA	2.0 (1.1 to 3.0)	24.4 (13.1 to 35.7)	5.3 (2.8 to 7.9)
Ohio	767.6 (753.0 to 782.2)	4.4 (2.6 to 6.3)	-33.1 (-53.1 to -13.0)	6.7 (3.9 to 9.4)	46.7 (10.3 to 83.1)	5.3 (1.0 to 10.0)

Abbreviations: CI = confidence interval; NA = not applicable

[a] Estimates from interrupted time series (ITS) models. All models utilize stepwise autoregression to control for serial autocorrelation. NA indicates that a post-period level change was no different from the pre-period value at the p<0.2 level.

© 2024 Christine PJ et al. *JAMA Network Open.*

eFigure. Number of X-Waivered Clinicians With a 30-Patient Limit Before and After Relaxation of the X-Waiver Training Requirements, May 2020 to May 2022[a]

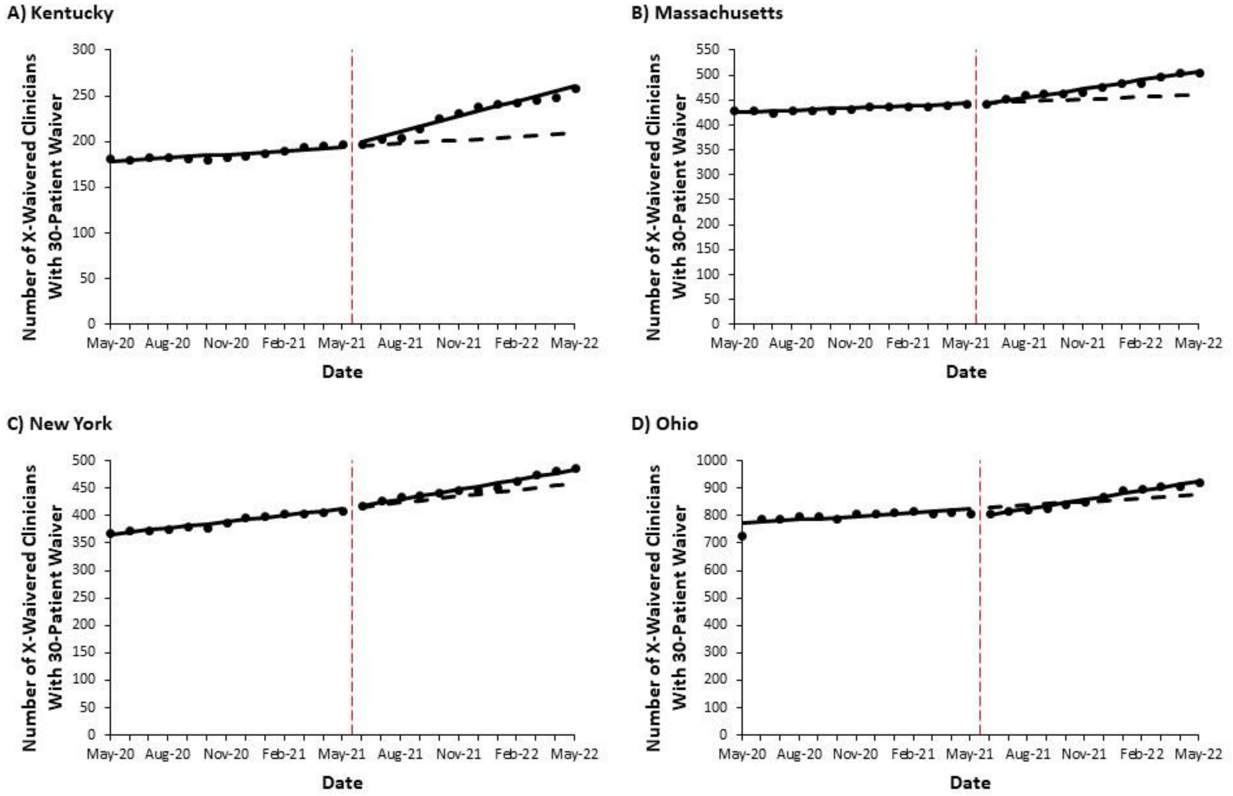

[a] Dashed red vertical line indicates the time of the X waiver training relaxation. Dashed black line indicates the predicted trend based on pre-period data. Solid black line indicates the modeled trend in the outcome accounting for level and linear trend changes due to the X waiver training relaxation. Points indicate observed data.

© 2024 Christine PJ et al. *JAMA Network Open.*

eTable 5. Interrupted Time Series Sensitivity Analysis for the Effect of the X-Waiver Training Relaxation on the Number of X-waivered Clinicians Prescribing Buprenorphine in Ohio and Massachusetts, Excluding Community-Months With Suppressed Data[a]

Outcome	Model Variable and 95% CI			
	**Intercept**	**Pre-Period Linear Trend**	**Post-Period Level Change**	**Post-Period Linear Trend Change**
Massachusetts				
Number of X-waivered clinicians prescribing buprenorphine to at least one patient	169.2 (161.4 to 177.1)	1.5 (0.5 to 2.5)	12.3 (1.5 to 23.1)	-1.7 (-3.2 to -0.3)
Ohio				
Number of X-waivered clinicians prescribing buprenorphine to at least one patient	318.3 (307.5 to 329.1)	3.8 (2.6 to 5.0)	NA	-2.7 (-4.8 to -0.5)

[a] Main models used in paper utilized multiple imputation for suppressed data in communities with fewer than five X-waivered clinicians who prescribe buprenorphine. A total of 48 (21.3%) and 85 (37.8%) community-months were excluded in Massachusetts and Ohio, respectively.

© 2024 Christine PJ et al. *JAMA Network Open.*

eTable 6. Number of Zip Codes With X-Waivered Clinicians in May 2021 and May 2022 and Training-Exempt X-Waivered Clinicians in May 2022[a]

State	Zip Codes with X-Waivered Clinicians in May 2021, No. (%)	Zip Codes with X-Waivered Clinicians in May 2022, No. (%)	Zip Codes with Training-Exempt X-Waivered Clinicians in May 2022, No. (%)	Zip Codes with Only Training-Exempt X-Waivered Clinicians in May 2022, No. (%)[b]
**Kentucky**	86/157 (54.7)	84/157 (53.5)	32/157 (20.4)	0/157 (0.0)
**Massachusetts**	27/47 (57.4)	26/47 (55.3)	14/47 (29.8)	0/47 (0.0)
**New York**	98/305 (47.8)	101/305 (49.3)	35/205 (17.1)	2/305 (1.0)
**Ohio**	108/240 (45.0)	108/240 (45.0)	33/240 (13.8)	0/240 (0.0)

[a] Includes all levels of X-waivers (30-patient, 100-patient, and 275-patient waivers).
[b] Corresponds to the number of zip codes with only training-exempt X-waivered clinicians and no other X-waivered clinician

© 2024 Christine PJ et al. *JAMA Network Open.*
