# Supplementary material for: Buprenorphine Prescribing Characteristics Following Relaxation of X-Waiver Training Requirements
Source: JAMA Netw Open. 2024 Aug 5;7(8):e2425999. doi: 10.1001/jamanetworkopen.2024.25999 (PMC11301557; doi:10.1001/jamanetworkopen.2024.25999)
Supplement: Supplement 2. — Data Sharing Statement [file jamanetwopen-e2425999-s002.pdf]

Data Sharing Statement

Christine. Buprenorphine Prescribing Characteristics Following Relaxation of X-Waiver Training Requirements. *JAMA Netw Open*. Published August 05, 2024. doi:10.1001/jamanetworkopen.2024.25999

Data
Data available: No

Additional Information
Explanation for why data not available: The data from the Healing Communities Study is not currently public as it is an ongoing randomized controlled trial. The data will eventually be made public once the trial is complete.
